# The *Vibrio vulnificus* stressosome is dispensable in nutrient-rich media

**DOI:** 10.1099/acmi.0.000523.v4

**Published:** 2023-07-13

**Authors:** Laura Cutugno, Jennifer Mc Cafferty, Jan Pané-Farré, Conor O'Byrne, Aoife Boyd

**Affiliations:** ^1^​ School of Natural Sciences, University of Galway, Galway, Ireland; ^2^​ Centre for Synthetic Microbiology (SYNMIKRO) and Department of Chemistry, Philipps-University Marburg, Marburg, Germany; ^3^​ School of Biological and Chemical Sciences, University of Galway, Galway, Ireland

**Keywords:** stress response, *Vibrio*, stressosome, survival

## Abstract

The stressosome is a protein complex that senses environmental stresses and mediates the stress response in several Gram-positive bacteria through the activation of the alternative sigma factor SigB. The stressosome locus is found in 44 % of Gram-negative *Vibrio vulnificus* isolates. However, *V. vulnificus* does not possess SigB. Nonetheless, in nutrient-limited media, the stressosome modulates gene transcription and bacterial behaviour. In this work, the expression of the stressosome genes was proven during stationary phase in nutrient-rich media and co-transcription as one operonic unit of the stressosome locus and its putative downstream regulatory locus was demonstrated. The construction of a stressosome mutant lacking the genes encoding the four proteins constituting the stressosome complex (VvRsbR, VvRsbS, VvRsbT, VvRsbX) allowed us to examine the role of this complex *in vivo*. Extensive phenotypic characterization of the ΔRSTX mutant in nutrient-rich media showed that the stressosome does not contribute to growth of *V. vulnificus *. Moreover, the stressosome did not modulate the tolerance or survival response of *V. vulnificus* to the range of stresses tested, which included ethanol, hyperosmolarity, hypoxia, high temperature, acidity and oxidative stress. Furthermore, the stressosome was dispensable for motility and exoenzyme production of *V. vulnificus* in nutrient-rich media. Therefore, in conclusion, although stressosome gene transcription occurs in nutrient-rich media, the stressosome neither has an essential role in stress responses of *V. vulnificus* nor does it seem to modulate these activities in these conditions. We hypothesise that the stressosome is expressed in nutrient-rich conditions as a sensor complex, but that activation of the complex does not occur in this environment.

## Data Summary

All data that have led to the conclusions drawn in this article are presented within the article and Supplementary Material.

## Introduction

*Vibrio vulnificus* is a human foodborne pathogen that causes severe human infections, with a fatality rate >50 % in the USA [1]. It populates coastal waters and is bio-accumulated by many bivalves, amongst which are oysters and other shellfish destined for human consumption [2]. Two infection pathways have been described for this pathogen: gastrointestinal and wound infection [2–4]. The former occurs when the bacteria are ingested via contaminated raw molluscs [1], while the latter is caused through contact of contaminated water with pre-existing wounds and this represents the most common infection route in the USA [5–8]. The fatality rate of the latter pathway is high, but the severity of the infection generally depends on underlying health conditions in the patient [2]. *V. vulnificus* is considered to be an opportunistic pathogen and severe infections have been observed in immunocompromised patients and those with pathological conditions that increase the level of iron in the blood, such as liver disease [9, 10]. The distribution of this pathogen is dependent on environmental factors, such as water temperature and salinity, which results in regionality of the cases of *V. vulnificus* infection [11, 12]. A key step to understanding and predicting the distribution of the microorganism and the occurrence of infection is elucidation of the mechanisms that constitute the bacterial stress response.

The bacterial stress response is the set of physiological changes and molecular mechanisms that a bacterium puts in place to survive changes in the environment that would otherwise be lethal [13]. The stress response is a fundamental step to persist in the environment and to guarantee a successful infection [14, 15]. It requires substantial changes in the physiology of the bacteria that often conflict with optimal growth and reproduction [16]. For this reason, it is essential to regulate the stress response and limit such changes to the right time and environmental conditions. To do so, bacteria have developed complex regulation mechanisms, often characterized by fast activation and, equally importantly, efficient inactivation systems. To optimally coordinate the stress response with the external environment, bacteria have evolved signalling complexes that sense the stress and integrate the signal to modulate the cell response. Amongst these is the bacterial stressosome, a 1.8 MDa complex that has been discovered and extensively characterized in the Gram-positive *Bacillus subtilis* [17–19]. In this organism and other Gram-positive bacteria, the stressosome has been found to activate the alternative sigma factor σ^B^ (SigB) following sensing of environmental stresses [20, 21]. SigB in its active form binds to the RNA polymerase (RNAP) and promotes the expression of hundreds of genes involved in the stress response [22].

Interestingly, the genetic locus encoding the stressosome proteins has been found in several phyla and in bacteria that do not possess SigB, such as *V. vulnificus* [23]. In this organism, the stressosome locus contains an upstream and a downstream module. The first is formed by the genes encoding the three stressosome proteins VvRsbR, VvRsbS and VvRsbT (equivalent to RsbR, RsbS and RsbT in *B. subtilis *) and the phosphatase VvRsbX (RsbX), with the latter encoding a putative regulatory output of the stressosome – a two-component system (TCS) most likely involved in c-di-GMP hydrolysis [24]. This locus has been identified in 44 % of sequenced genomes and its expression has been proven in both natural and laboratory conditions [25–28]. The haem-binding globin domain at the N-terminal of VvRsbR and the biochemical characterization of the corresponding *Vibrio brasiliensis* proteins suggested a potential role for the stressosome in surviving or tolerating anaerobic conditions [28, 29]. More recently, studies of the role of the *V. vulnificus* stressosome in nutrient-limited conditions have shown that it regulates protein expression, glucose metabolism and motility [26, 30]. This role would be relevant both for the persistence of *V. vulnificus* in the environment and for survival during the infection process. For this reason, this work focused on characterization of the *in vivo* role of the stressosome in this marine pathogen during growth in rich media, to complement the previous studies in chemically defined minimal media [28]. This characterization confirmed that the stressosome genes are transcribed in LB+2.5 % NaCl (LBN), a rich media commonly used to culture this bacterium, and focused on the effects of the stressosome on the growth and stress response of *V. vulnificus *. Moreover, due to its potential role in regulating the levels of c-di-GMP, motility and other virulence traits were analysed to elucidate the role of the stressosome in the pathogenesis of *V. vulnificus *.

## Methods

### Strains, plasmids and growth conditions

The wild-type clinical strain *V. vulnificus* CMCP6 [31] and the ΔRSTX stressosome mutant derivative of CMCP6 were the focus of analysis in this study [28, 30]. A rifampicin-resistant derivative of CMCP6 (Rif^R^3 encoding RpoB^H526Y^) [32] was used for the construction of a rifampicin-resistant stressosome mutant. *V. vulnificus* was cultured in lysogeny broth medium with an additional 0.4 M NaCl (LBN) at 30 °C or 37 °C, as specified. Overnight cultures were grown in 2 ml broth in 15 ml bacterial culture tubes, at 37 °C with agitation at 150 r.p.m. Specific growth conditions, different from those indicated above, are described in the appropriate sections. All chemicals and reagents were supplied by Sigma-Aldrich, unless indicated otherwise. All molecular biology kits were utilized according to the manufacturer’s instructions.

### RNA extraction and one-step RT-PCR analysis

For RNA extraction, *V. vulnificus* wild-type CMCP6 was grown overnight in LBN or chemically defined medium [CDM: 9.94 mM Na_2_HPO_4_, 10.03 mM KH_2_PO_4_, 0.81 mM MgSO_4_∙7H_2_O, 9.35 mM NH_4_Cl, 856 mM NaCl, 0.75 µM FeCl_3_, 7.5 mM α-D(+)-glucose] at 37 °C. One volume of bacterial culture containing approximately 0.2 OD_600_ of cells was mixed with two volumes of RNAprotect (Qiagen) and processed according to the manufacturer’s instructions. The pellet was used for RNA extraction within 24 h of treatment with RNAprotect. The pellet was resuspended in 200 µl TE buffer (30 mM TrisHCl, 1 mM EDTA, pH 8.0)+15 mg ml^−1^ lysozyme+10 µl ready-to-use Qiagen proteinase K (20 mg ml^−1^) and incubated for 10 min at room temperature to achieve cell lysis. RNA extraction on the lysate was performed with the RNeasy Mini kit (Qiagen). The total RNA was eluted in 30 µl RNase-free water and traces of DNA were removed using the TURBO DNA-free kit (Life Technologies). The concentration and quality of the extracted RNA were assessed with a NanoDrop spectrophotometer and only samples with a 260/280 ratio ≥1.9 were used for the RT-PCR protocol. RNA integrity was verified on a 1.5 % agarose gel. To assess the presence of the target mRNA a one-step RT-PCR protocol was performed, using the Qiagen OneStep RT-PCR kit. Briefly, the procedure consisted of two consecutive steps: one of reverse transcription (RT) at 50 °C for 30 min and one of traditional PCR. PCR primers (Table 1) were designed using Primer-blast [33]. An RT negative control that skipped the step at 50 °C was included to confirm the absence of DNA contamination. A PCR negative control in which the template was substituted with PCR-grade water was included for each pair of primers. The gene *tuf* (elongation factor Tu) was used as endogenous control. PCR products were visualized on a 1.2 % agarose gel.

**Table 1.** Primers for co-transcription experiments of the stressosome locus genes in *V. vulnificus* CMCP6 wild-type

Gene	Sequence (5’->3’)
***tuf* (VV1_1203**)	Forward: AAGTTTACGGCGGTGCTGCT Reverse: CGTAGTGGCGAGCTGGAGTG
***VvrsbRS***	Forward: GGTTCGTACCAATGCCACCT Reverse: TGGTCGATTTCACCGCATCT
***VvrsbST***	Forward: GGGGGTTGTGATTGGCCTTA Reverse: TGCGTACTTCACCACGTTCA
***VvrsbTX***	Forward: CCGGGGATCCACAACATTGA Reverse: CCAGAGACATATTCGCCGCT
***VvrsbXD1***	Forward: GTGGTCGGAGAATTGTTGCC Reverse: TAGCCAAAGGCTTGCAGGAT
***VvD1D2***	Forward: CATTAGGCAGACCCAAGCCA Reverse: TGAGACTGGCACGCAGAAAT
***VvrsbR***	Forward: GGCTCAGAAACACCCCTGAA Reverse: AGTTGGCAATGGTAAGCCGA
***VvD1***	Forward: GGAAGAGGCGGTTGAAGTGA Reverse: CATGCTGCTTGGGGGTAAGA

### Construction of the *V. vulnificus* ΔRSTX knock-out mutant

The construction of a knock-out mutant lacking the upstream module of the stressosome locus (* V. vulnificus* ΔRSTX) was first achieved using a classical conjugation protocol with the rifampicin-resistant Rif^R^3 strain of *V. vulnificus* (Rif^R^) and *Escherichia coli* SM10λpir (pDS_*ΔRSBRSTX*). The use of rifampicin-resistant *V. vulnificus* has been previously employed [34, 35] to counter-select the donor strain after conjugation. pDS_*ΔRSBRSTX* carries the knockout allele with a deletion from nucleotide 4 of *VvrsbR* to nucleotide 577 of *VvrsbX* in the pDS132 suicide vector [28, 30, 36, 37]. Biparental conjugations with *E. coli SM10λpir (pDS_ΔRSBRSTX*) were performed to introduce the allele into Rif^R^ *V. vulnificus* and selection of first recombinants was performed on LBN agar containing 5 µg ml^−1^ chloramphenicol+50 µg ml^−1^ rifampicin.

Subsequently, second recombinants were selected on LBN agar containing 10 % sucrose and then screened by PCR with primers RSTX_For and RSTX_Rev [30]. Bacteria that contained the gene of the expected shortened length were designated the *ΔRSBRSTX* mutant strain. One putative mutant was selected and whole-genome sequencing (WGS) analysis was performed to confirm the mutation. In addition, the *V. vulnificus* CMCP6 and the Rif^R^ strain were also sequenced. Genomic DNA was extracted using the Wizard Genomic DNA Purification kit (Promega) and sequenced by MicrobesNG (Birmingham, UK) using Illumina technology. Average read lengths were between 168 and 645 nucleotides for each sample and average fold coverage was between 52 and 1162. BreSeq was used to call base substitution mutations with read alignment evidence using consensus mode, with a mutation E-value cut-off of 10 and a frequency cut-off of 0.8 (80 %).

To avoid the use of a rifampicin-resistant strain of *V. vulnificus* that exhibits pleiotropic effects [32] that interfere with the phenotypic characterization of the stressosome mutant, we analysed a *ΔRSTX* mutant generated via conjugation of *V. vulnificus* CMCP6 and a DAP-auxotrophic strain of *E. coli* β2163 carrying *pDS_ΔRSBRSTX* [28, 30, 36].

### Growth characterization in LBN

Growth was assessed in atmospheric and reduced oxygen conditions at 30 °C in LBN. Precultures were grown aerobically for 16–18 h in 2 ml LBN at 37 °C and then diluted in LBN to an initial OD of 0.01. For atmospheric oxygen conditions 200 µl cell suspension was inoculated in a microtitre plate and incubated at 30 °C. To achieve oxygen-depleted growth conditions a higher volume of cell suspension (approximately 300 µl) was used to completely fill the microtitre well and the plate was sealed with a sterile adhesive plastic film before incubation. To assess growth at 42 °C, the plate was set up as described and incubated at 37 °C for 1 h and then at 42 °C. At least three biological replicates were used for each strain and each of them was assessed in two technical replicates. The plates were statically incubated in a Sunrise microtitre plate reader at the specified temperature and the OD_595_ was measured every 30 min for 24 h.

### Stress survival assays

The survival of both *V. vulnificus* wild-type and the ΔRSTX strain was assessed in LBN in the presence of different stresses. Overnight cultures were grown at 37 °C in 2 ml LBN for no longer than 18 h. The cultures were then diluted to OD 0.1 in 2 ml of the appropriate stress media and incubated at 30 °C (except the samples tested for temperature stress). The stresses (and media compositions) were: ethanol stress [LBN+10 % ethanol (v/v)], oxidative stress (LBN+2 mM H_2_O_2_), acid stress (LBN pre-acidified to pH 4 with HCl) and temperature stress (45 °C in a temperature-controlled block). One hundred microlitre samples were withdrawn at different time points, serially diluted and spread on agar, and colony-forming units (c.f.u.) were counted after overnight incubation. At least two biological replicates were tested for each strain and stress condition, each one assessed in two technical replicates for the plate counting.

### Stress tolerance assays

To analyse the ability of *V. vulnificus* wild-type and the ΔRSTX strain to tolerate stress and to grow in non-lethal stress conditions, we tested the growth of the two strains on LBN agar supplemented with several stressors. Overnight cultures were grown at 37 °C in 2 ml LBN for no longer than 18 h and then diluted to OD 1 in LBN broth. Cell suspensions were then 10-fold serial diluted up to 10^−7^ and 3 µl of each dilution was spotted on the appropriate LBN agar plate and incubated at 30 °C. The stresses (and media compositions) were: oxidative stress (LBN agar+0.5 mM H_2_O_2_) and osmotic stress (LB agar+0.8 M NaCl). To test growth ability in anaerobic conditions, the plates were incubated in a 2.5 l anaerobic jar in the presence of an Oxoid AnaeroGen 2.5 l sachet that generates an atmosphere with <1 % oxygen, according to the manufacturer. Pictures of the plates were taken after 24 and 48 h growth.

### Motility assay

In order to evaluate the effect of the stressosome mutation on the motility of *V. vulnificus *, the two strains were tested for swimming on rich motility agar plates (10 g tryptone, 20 g NaCl and 3.35 g agar l^−1^). Overnight cultures were grown in 2 ml LBN broth at 37 °C. A sterile metal wire was then immersed in the cell suspension and used to pierce the motility plate. Plates were incubated at 30 or 37 °C and the motility zone was measured after 16 h. At least three biological replicates were tested for each strain.

### Exoenzyme production

The wild-type and the ΔRSTX strains were tested for the production of two exoenzymes, haemolysin and protease. The strains were grown overnight in 2 ml LBN broth at 37 °C. The cell suspension was then inoculated on LBN agar plates containing 5 % (v/v) defibrinated sheep blood (Thermo Fisher Scientific) for haemolysin assay and 1 % (w/v) skim milk for protease assay. The plates were incubated at 37 °C and pictures were taken after 48h and 24 h, for the haemolysin and protease tests, respectively.

### Cross-protection assay

Cross-protection experiments were performed as previously described [38] to assess the effects of nutrient downshift on temperature survival in *V. vulnificus* wild-type and ΔRSTX. The data values presented are the average of two technical replicates. Briefly, the strains were grown overnight in 2 ml LBN at 37 °C. Cultures were then diluted in 2 ml of fresh LBN to OD_600_ 0.05 and grown to mid-log phase (OD_600_ 0.4–0.6). Cell cultures were then diluted 1 : 100 in CDM in the absence of carbon source (9.94 mM Na_2_HPO_4_, 10.03 mM KH_2_PO_4_, 0.81 mM MgSO_4_∙7H_2_O, 9.35 mM NH_4_Cl, 856 mM NaCl, 0.75 µM FeCl_3_). Cell suspensions were incubated at 37 °C and 1 ml was withdrawn at different time points (0, 0.5, 1, 2, 4 and 24 h) to test survival at high temperature. Plate counting was performed at each time point, before and after 1 h of incubation at 45 °C. Mid-log phase cells diluted in LBN rather than CDM were used as a control, to confirm temperature sensitivity before nutritional downshift.

## Results

### The stressosome locus is expressed in rich media with co-transcription of all modular genes

Previous reports have shown that *VvrsbR, VvrsbS, VvrsbT* and the downstream TCS are expressed in artificial seawater (ASW) [26] and, at the protein level, expression has been confirmed in minimal medium [28], but little information was available on the expression and possible role of the stressosome in *V. vulnificus* during growth in rich media [39]. Moreover, although the locus is predicted to be an operon, due to close proximity (sometimes overlapping) of the genes to one another, no proof of co-transcription has been provided to date. To address these two questions, the presence of single transcripts and co-transcripts was investigated in cells growing in LBN at stationary phase through RNA extraction and non-quantitative reverse-transcriptase PCR (RT-PCR).

**Fig. 1.**
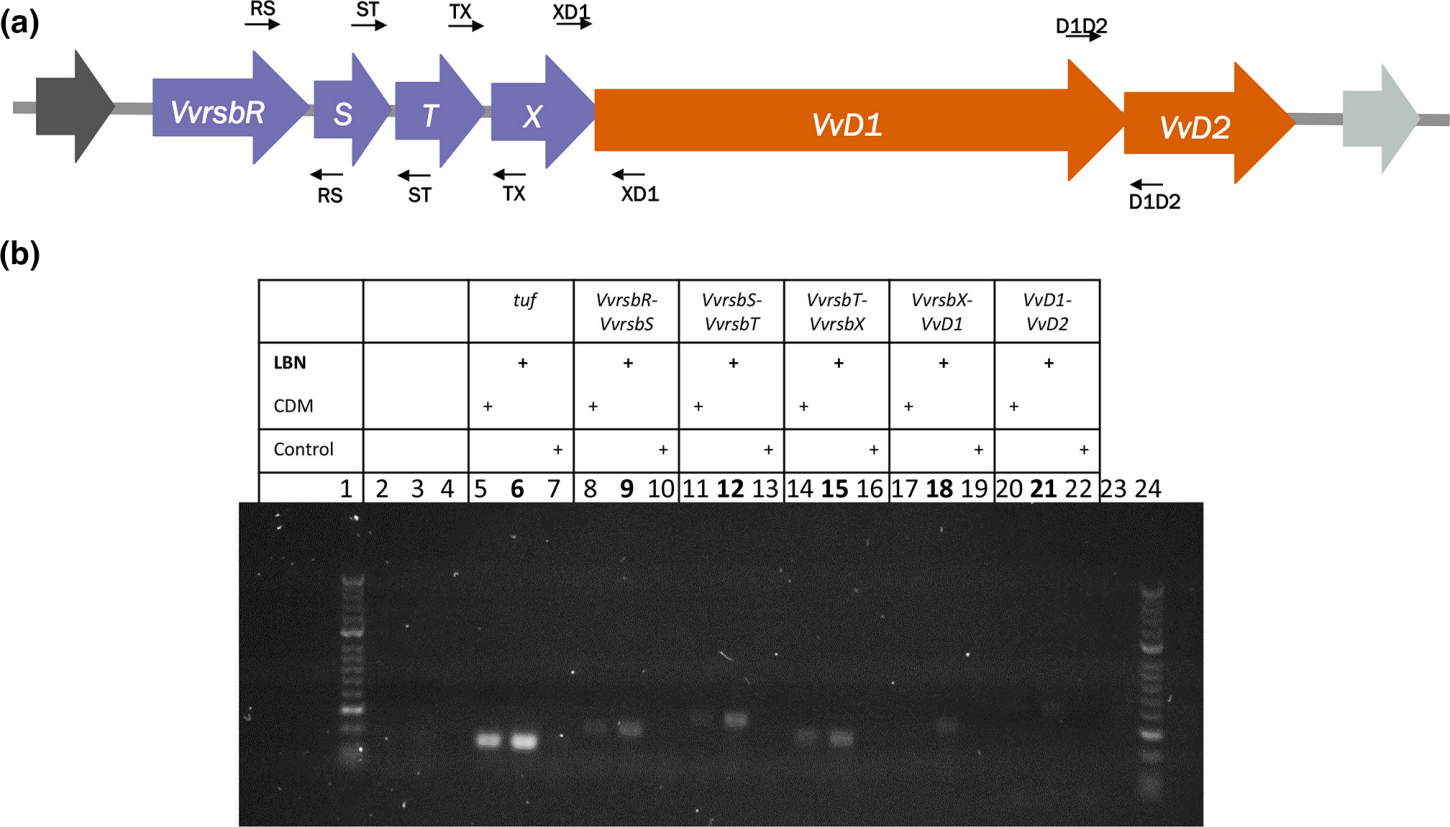
Co-transcription of stressosome locus genes in *V. vulnificus *. (**a**) The genetic stressosome locus in *V. vulnificus* CMCP6 wild-type, formed by the upstream module (*VvrsbR, VvrsbS, VvrsbT and VvrsbX*) in purple and the downstream module (*VvD1* and *VvD2*) in orange. The primers used for the co-transcription analysis are indicated at the top (forward primers) and at the bottom (reverse primers) of each gene. (**b**) Co-transcription analysis of the stressosome locus genes in chemically defined mediumChemically Defined Medium (CDM) and LBN. Electrophoresis analysis of the products of the RT-PCR performed on stationary phase cells growing in CDM or LBN at 37 °C. 1. HyperLadder 50 bp (Bioline); 2, *tuf* RT negative control – CDM; 3, *tuf* RT negative control – LBN; 4, empty lane; 5, *tuf* gene – CDM; 6, *tuf* gene – LBN; 7, *tuf* PCR negative control; 8, *VvrsbR-VvrsbS* co-transcript – CDM; 9, *VvrsbR-VvrsbS* co-transcript – LBN; 10, *VvrsbR-VvrsbS* PCR negative control; 11, *VvrsbS-VvrsbT* co-transcript – CDM; 12, *VvrsbS-VvrsbT* co-transcript – LBN; 13, *VvrsbS-VvrsbT* PCR negative control; 14, *VvrsbT-VvrsbX* co-transcript – CDM; 15*, VvrsbT-VvrsbX* co-transcript – LBN; 16, *VvrsbT-VvrsbX* PCR negative control; 17, *VvrsbX-VvD1* co-transcript – CDM; 18, *VvrsbX-VvD1* co-transcript – LBN; 19, *VvrsbX-VvD1* PCR negative control; 20, *VvD1-VvD2* co-transcript – CDM; 21, *VvD1-VvD2* co-transcript – LBN; 22, *VvD1-VvD2* PCR negative control; 23, empty lane; 24, HyperLadder 50 bp (Bioline).

First, the presence of the transcripts of the *VvrsbR* and *VvD1* genes was confirmed in cells growing in LBN, demonstrating that the locus is expressed in nutrient-rich media (Fig. S1, available in the online version of this article). Moreover, the detection of RT-PCR products with the use of primers in the proximity of intergenic regions at the 3′ and 5′ ends of adjoining genes (Fig. 1a) indicated the presence of co-transcripts between each pair of genes within each module and between *VvrsbX* and *VvD1* (Fig. 1b), showing that the upstream and downstream module genes are co-transcribed in CDM and LBN. The expression of the stressosome and its downstream module genes in LBN suggested a role of this complex in rich media, while their proximity on the chromosome and the suggested presence of a common mRNA support the idea of the two modules being functionally related to each other.

### Phenotypic characteristics of the stressosome mutant were overshadowed by the pleiotropic effects of the Rif^R^ allele

Phenotypic characterization of the ΔRSTX stressosome mutant constructed in the *V. vulnificus* rifampicin-resistant background was performed in order to identify the *in vivo* role of the stressosome in this human pathogen. The mutant was tested for growth, stress survival and tolerance, and for the main virulence characteristics, and compared to the *V. vulnificus* CMCP6 wild-type strain. The use of the rifampicin-resistant parental strain as control allowed us to identify possible effects of the rifampicin resistance and validate the use of the classical mutagenesis protocol when downstream applications include stress response and virulence characterization of the mutants. In some cases, the mutant showed differences compared to the wild-type strain but all were ascribable to pleiotropic effects of the rifampicin-resistant parental strain. In particular, reduced motility, ethanol survival and hyperosmotic stress tolerance were observed (Fig. S2). However, the same effects were observed, to the same extent, in the rifampicin-resistant parental strain, in line with previously published work that demonstrated the pleiotropic effects of the rifampicin-resistant variant (RpoB^H526Y^) [32]. This is an indication that the construction of the stressosome mutant in a Rif^R^ strain might not allow the detection of phenotypes caused by the *VvrsbRSTX* deletion, when these overlap with effects associated with rifampicin-resistant alleles. Based on this, a different mutagenesis protocol was optimized, resulting in the successful introduction of the ΔRSTX allele into a wild-type background. This allowed a direct comparison of the mutant with the wild-type and extensive characterization of the role of the stressosome in LBN medium. The same strain was used in recently published work, demonstrating the role of the stressosome in minimal medium [28], thus allowing us to directly compare effects of the mutation in different growth conditions.

### Knockout mutation of the stressosome does not influence growth of *V. vulnificus* in LBN, at various oxygen concentrations and temperatures

In order to test the physiological role of the stressosome in *V. vulnificus *, a stressosome mutant lacking the upstream module (* V. vulnificus* ΔRSTX) was successfully constructed and used for phenotypic characterization in rich media in comparison to the wild-type strain. Firstly, growth characterization of the two strains was performed in LBN at 30 and 37 °C in aerobic conditions to eliminate any possible influence of differential growth rate on the phenotypic characteristics analysed. The two strains grew with similar kinetics in these conditions (Fig. S3).

**Fig. 2.**
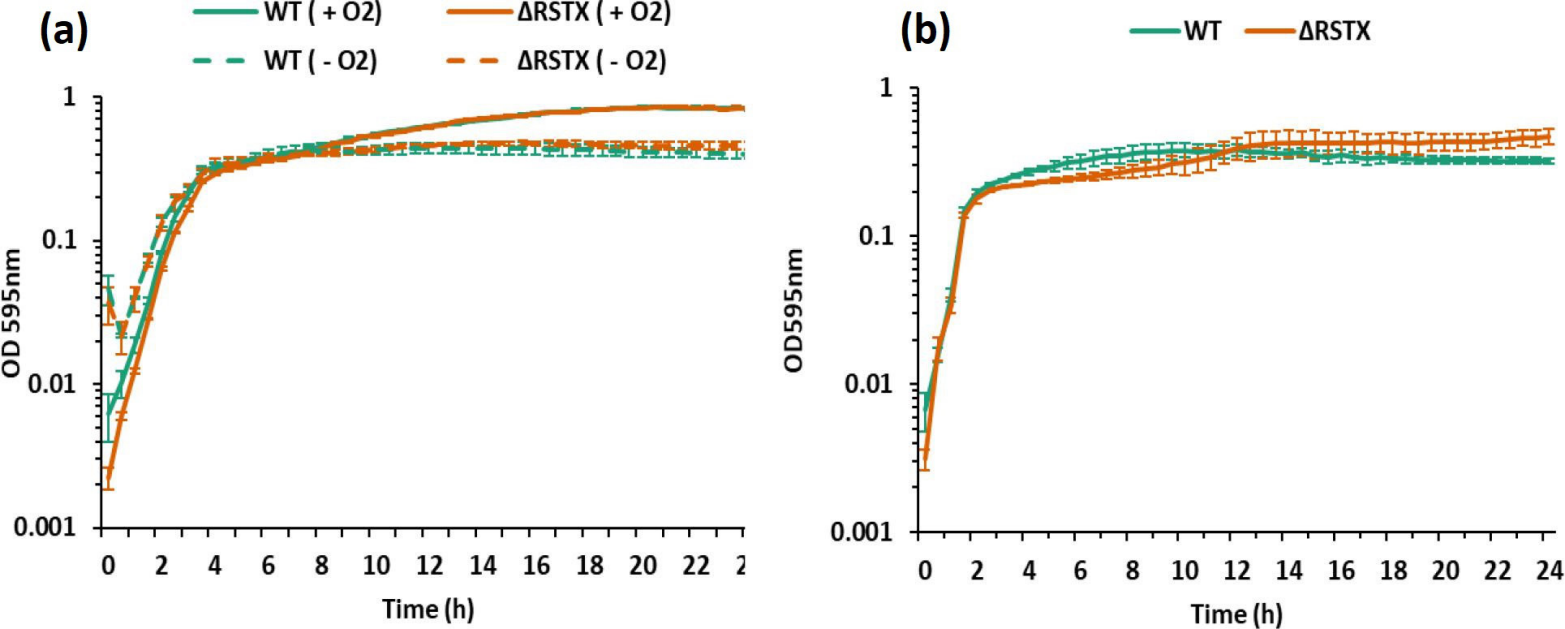
Growth characterization of *V. vulnificus* CMCP6 wild-type (green) and ΔRSTX (orange) in LBN. OD_595_ was measured every 30 min for 24 h. Each curve is the mean±sd of three biological replicates. (**a**) Growth in LBN at 30 °C in the presence of oxygen (continuous line) and in reduced oxygen conditions (dashed line). (**b**) Growth in LBN at 42 °C after 1 h growth at 37 °C.

Next, growth characterization of the two strains was performed in LBN at 30 and 37 °C in aerobic and O_2_-depleted conditions. *V. vulnificus* experiences variation in O_2_ levels both in the environment and in the human host [27]. Moreover, the *V. brasiliensis* stressosome has been shown to bind O_2_ *in vitro* [29]. For these reasons, growth curves in aerobic and reduced O_2_ conditions at 30 °C (Fig. 2a) were compared. Interestingly, oxygen depletion did not cause any growth rate variation for the wild-type, but only a reduction in the overall biomass accumulation, indicating good adaptation of *V. vulnificus* to hypoxia. Moreover, the growth profiles of the mutant and wild-type were comparable, indicating no role of the stressosome in adaptation to low oxygen levels in this growth condition.

During infection of the human host, *V. vulnificus* often faces not only reduced oxygen levels but also increasing temperatures due to the occurrence of fever in the patient [40]. For this reason, the ability of the two strains to grow at 42 °C was analysed after a brief time of growth at 37 °C (Fig. 2b). In this experimental setup, both the wild-type and the mutant strain showed slightly reduced growth when compared to the optimal growth temperature, but no differences were observed between the two strains. These growth experiments demonstrate that the presence of the stressosome does not influence growth rates in LBN, and nor is it required for the processes of adaptation to low oxygen levels or high temperatures in nutrient-rich media.

### The stressosome mutation does not alter the ability of *V. vulnificus* to survive lethal environmental stress in LBN

**Fig. 3.**
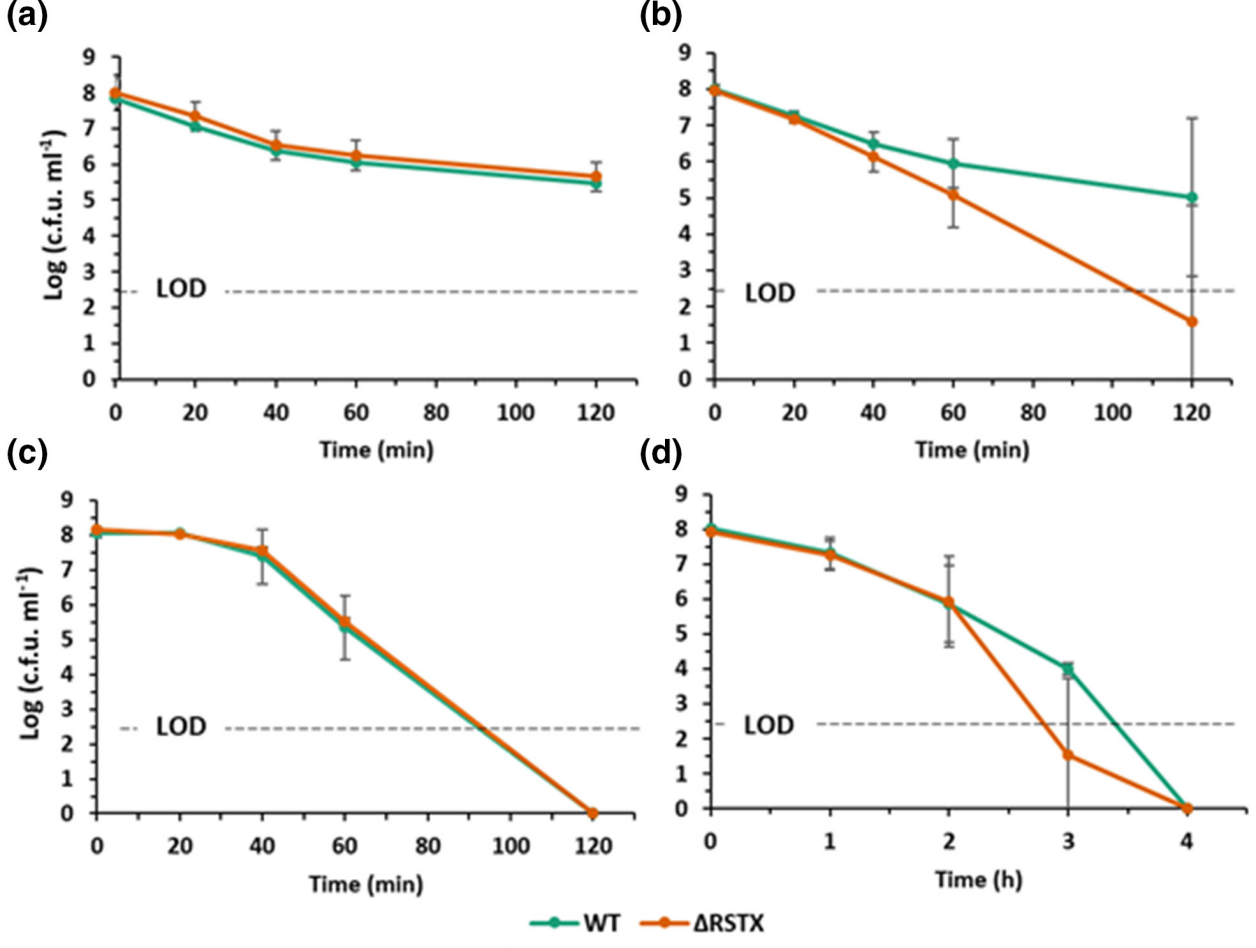
Stress survival of *V. vulnificus* CMCP6 wild-type (green) and ΔRSTX (orange) in LBN. (**a**) Survival assay in LBN+10 % ethanol (v/v). (**b**) Survival assay in LBN+2 mM H_2_O_2_. (**c**) Survival assay in LBN at pH 4. For these three analyses the strains were incubated at 30 °C and survival was assessed through colony counting at five time points (0, 20, 40, 60 and 120 min). (**d**) Survival assay in LBN at 45 °C. The strains were incubated at 45 °C and survival was assessed through colony counting at five time points (0, 1, 2, 3 and 4 h). For each time point, survival was plotted as log_10_ (c.f.u. ml^-1^). The reported values are the mean±sd of three biological replicates. Student’s *t*-test was performed comparing mutant strains to WT and all *P* values were >0.05. Limit of detection (LOD) is indicated by a black dotted line.

In Gram-positive bacteria, such as *Listeria monocytogenes* and *B. subtilis *, the stressosome is part of the signalling hub that ultimately leads to the activation of the alternative sigma factor σ^B^ [24]. This controls the transcription of hundreds of genes involved in the stress response and contributes to the survival of the bacteria in harsh environmental conditions [16, 41, 42]. To study a possible role of the stressosome in the survival of *V. vulnificus* to lethal stresses, survival assays in the presence of a range of different stressors were performed using the wild-type *V. vulnificus* CMCP6 and ΔRSTX (Fig. 3). We tested several stresses, including 10 % ethanol (Fig. 3a), 2 mM H_2_O_2_ (Fig. 3b), pH 4 (Fig. 3c) and high temperature (45 °C) (Fig. 3d). Response mechanisms to these stresses have only been partially elucidated in *V. vulnificus* [43–45]. The two strains equally survived the tested stresses. In particular, no differences in viability were observed in 10 % ethanol (Fig. 3a) or at pH 4 (Fig. 3c), confirming that the use of the Rif^R^ parental strain was the cause of the previously observed difference. A faster death was occasionally observed in the presence of H_2_O_2_ (Fig. 3b) or after the shift to 45 °C (Fig. 3d). This might have a biological meaning and indicate a potential modulatory involvement of the stressosome or simply be due to overall higher variability in these stress conditions.

Higher temperatures (48 and 50 °C) and hyperosmolarity (LBN+1.2 M NaCl) were also tested, but these conditions were too harsh or too mild, respectively, to reveal differences between the strains. These temperatures caused the complete death of both strains in less than 20 min, making it challenging to perform a time series experiment. The presence of an additional 1.2 M NaCl, in contrast, did not cause bacterial death. These results indicate that in our experimental conditions the stressosome does not contribute to the survival of *V. vulnificus* against environmental stresses and its deletion does not affect the ability of this human pathogen to survive several stresses that can be encountered both in the environment and in the human host.

**Fig. 4.**
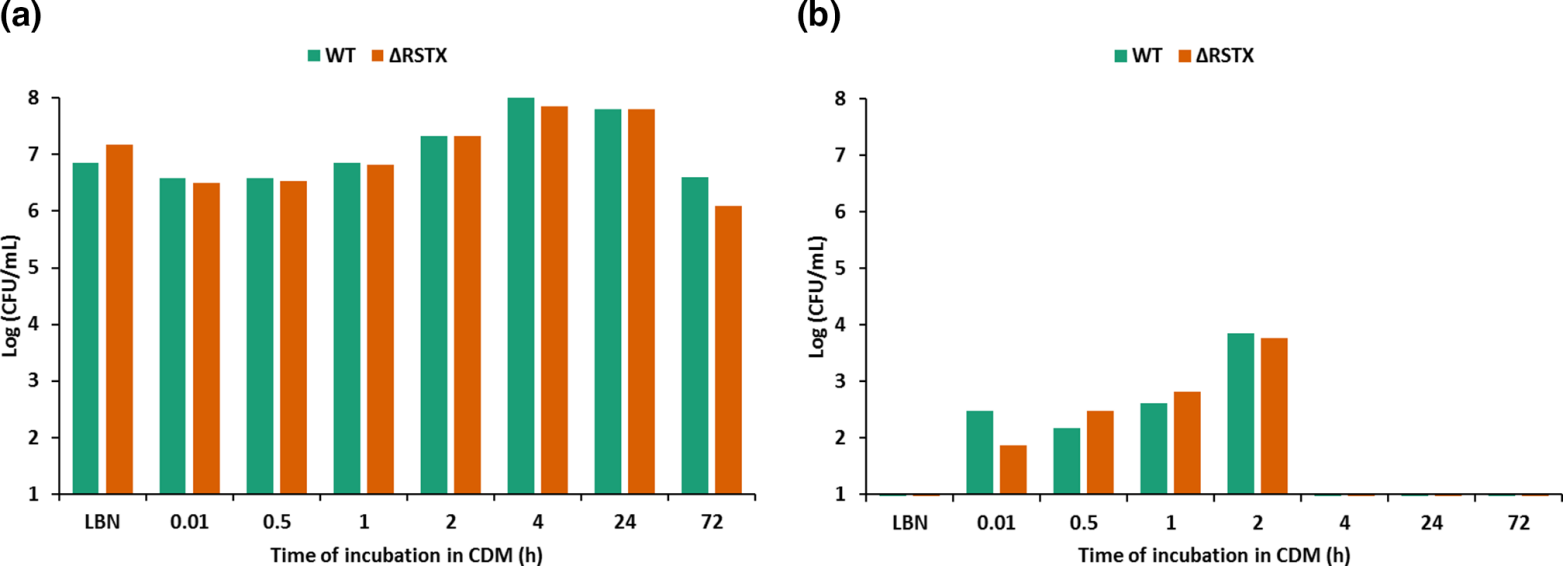
The cross-protection mechanism in *V. vulnificus* CMCP6 wild-type (green) and ΔRSTX (orange). (**a**) Cell viability upon nutrient downshift from LBN to CDM. The strains were incubated at 37 °C and survival was assessed through colony counting before the shift from LBN to CDM (LBN) and at six subsequent time points (0, 0.5, 1, 2, 4 and 24 h). (**b**) Survival in LBN at 45 °C for 1 h. Survival at high temperature was assessed through colony counting after 1 h incubation at 45 °C. The data values are the average of two technical replicates.

Concerning stress survival, several cases of cross-protection mechanisms have been described in *Vibrio* spp. [46] and in *V. vulnificus* specifically [38, 47], where pre-exposure to sub-lethal stresses (such as nutrient downshift) causes a general stress adaptation response that results in an increase in survival to subsequent exposure to lethal conditions. In this work, the role of the stressosome in the general stress adaptation response was assessed by measuring survival at high temperature following nutrient downshift – a methodology well described in *V. vulnificus* [38]. We confirmed that the shift from a rich to a chemically defined media, lacking a carbon source, rapidly caused a transient resistance to lethal heat stress (1 h at 45 °C), but no differences were observed between the wild-type and the stressosome mutant (Fig. 4). The cross-protective adaptive response occurred within 5 min following nutrient downshift and persisted for 2 h, after which time the adaptive response was resolved and normal responses returned. These experiments suggest that the stressosome does not regulate stress survival, either directly or indirectly through cross-protection adaptive mechanisms.

### The stressosome does not modulate stress tolerance in *V. vulnificus* in nutrient-rich media

**Fig. 5.**
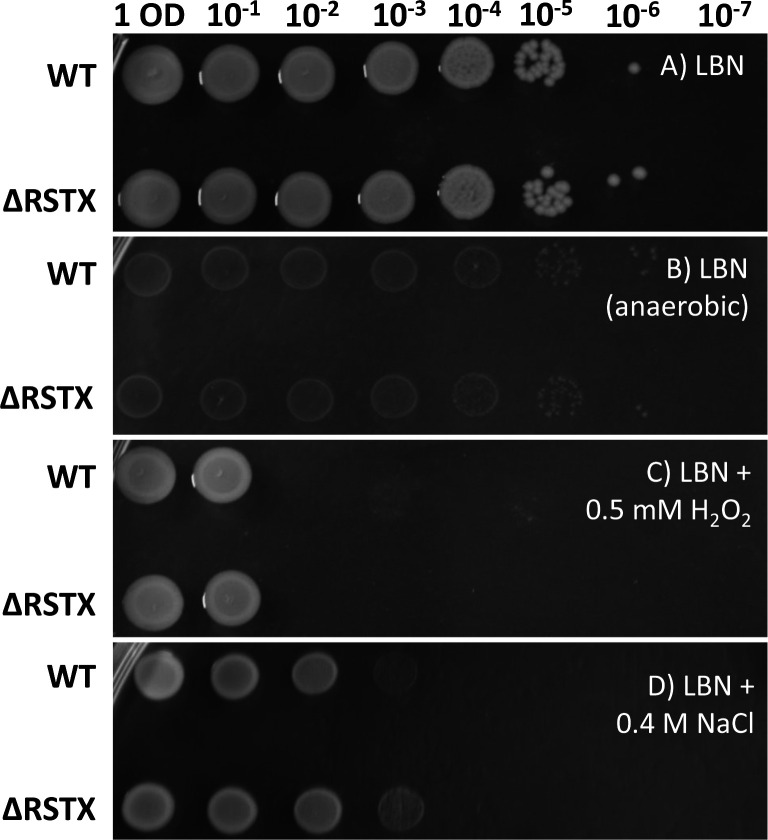
Growth assessment in stress conditions of *V. vulnificus* CMCP6 wild-type and ΔRSTX in LBN. The strains were first diluted to OD_600_=1 and then 10-fold serial dilutions were performed up to 10^−7^ and each dilution was spotted on agar plates and incubated at 30 °C for 24 h. (**a**) LBN agar (LB agar+0.4 M NaCl) in atmospheric condition. (**b**) LBN agar in oxygen-depleted condition. (**c**) LBN agar+0.5 mM H_2_O_2_ in atmospheric condition. (**d**) LB agar+0.8 M NaCl in atmospheric condition. The images shown are representative of at least two biological replicates.

Bacterial stress response refers not only to survival to extreme lethal stresses but also to stress tolerance mechanisms that allow the microorganisms to grow in the presence of milder stresses. This is essential to allow a bacterium or a bacterial community to reproduce and grow in the environment, which must be considered as a dynamic system in which small changes happen continuously due to natural fluctuations, human intervention and the presence of other organisms and micro-organisms [48]. To test the ability of our strains to tolerate, adapt and grow in the presence of mild stresses, we analysed their growth on LBN agar in the presence of different stressors. Amongst these, we tested 0.5 mM H_2_O_2_, anaerobiosis and hyperosmotic stress (LB+0.8 M NaCl) (Fig. 5). Various degrees of growth effects were observed in the presence of these stresses for the wild-type, with 0.5 mM H_2_O_2_ causing the strongest inhibitory effect with a reduction of 5 log in c.f.u. numbers when compared to the LBN control. The presence of extra salt strongly reduced the growth of *V. vulnificus *, while anaerobiosis only caused an effect on the amount of biomass present (i.e. smaller colonies indicative of a reduced replication rate) but no changes in the ability to initiate growth (i.e. equal number of colonies). The stressosome mutant strain suffered comparable growth reductions to those of the wild-type in all tested conditions.

To investigate the modulation of stress responses by the stressosome in reduced O_2_ conditions, the growth of the wild-type and ΔRSTX strains in conditions of combined NaCl and anaerobic stress was analysed (Fig. S4). The growth of both strains was greatly impaired in these conditions, as compared to growth in high salinity or reduced oxygen alone, but there was no difference in growth and stress tolerance between the two strains. This confirms that the stressosome is not involved in the stress response of *V. vulnificus* in nutrient-rich media.

### The stressosome does not affect motility and exoenzyme production

The previous experiments indicated that the stressosome did not play a major role in regulating growth and stress response in *V. vulnificus* in nutrient-rich media, in terms of stress survival, stress adaptation and stress tolerance. We next investigated another fundamental aspect of the life cycle of a human pathogen: virulence characteristics. Virulence includes a wide range of physiological processes from motility to biofilm formation, through exoenzyme and toxin production, and it is intrinsically connected to both growth and stress response [49, 50].

**Fig. 6.**
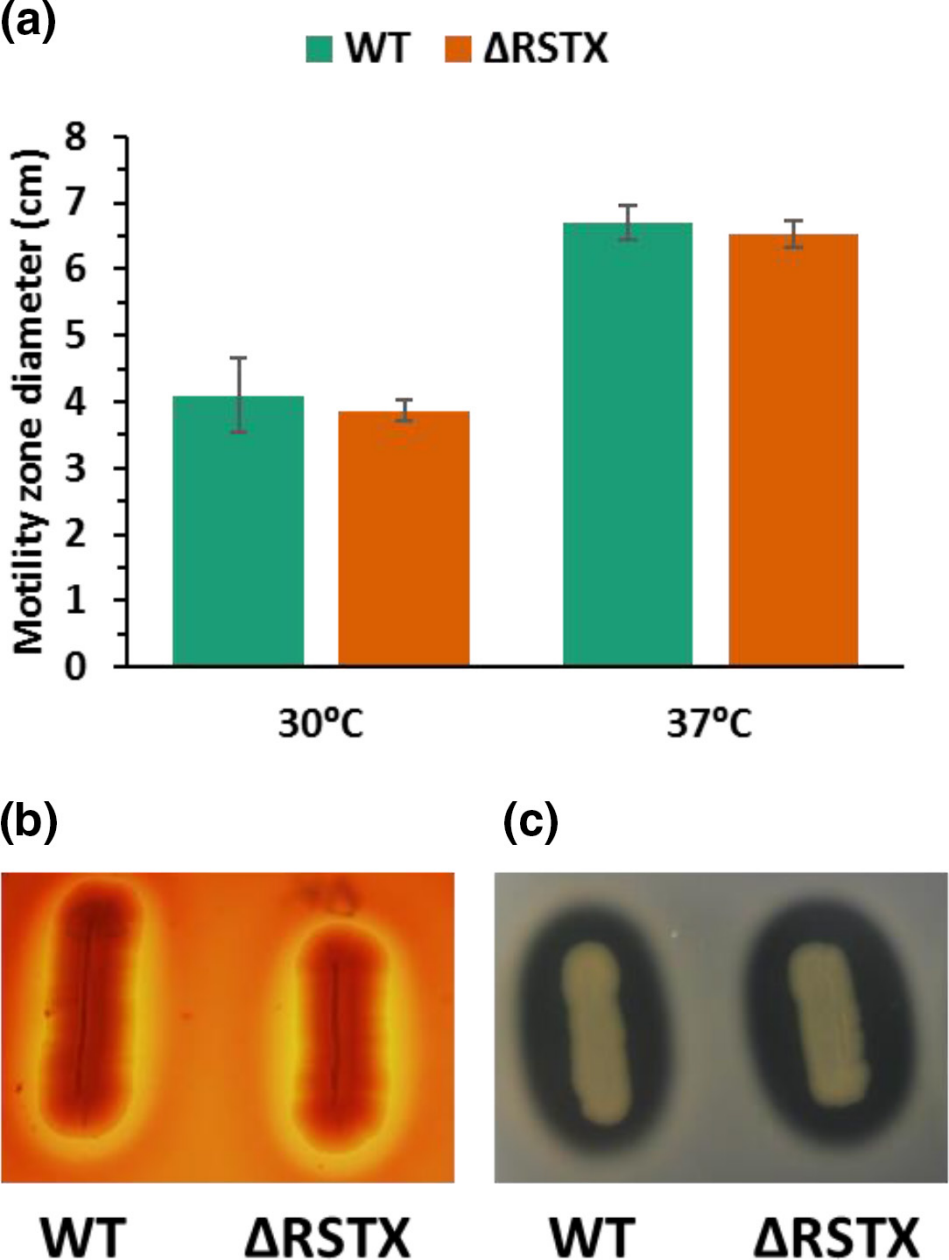
Motility and exoenzyme production of *V. vulnificus* CMCP6 wild-type (green) and ΔRSTX (orange) in rich media. (**a**) Motility assay on tryptone motility plates. The strains were stabbed on motility agar plates and incubated at 30 and 37 °C. The motility zone was measured after 16 h incubation. The reported values are the mean of three biological replicates. Student’s *t*-test was performed comparing mutant strain to WT and *P* values were >0.05. (**b**) Haemolysin production was assessed on LBN agar+5 % sheep blood (v/v). Images were acquired after 48 h incubation at 37 °C. (**c**) Protease production was analysed on LBN agar+1 % skimmed milk. Images were acquired after 24 h incubation at 37 °C.

In this work, we assessed the ability of both strains to swim on tryptone motility agar plates and their ability to produce haemolysin and protease exoenzymes (Fig. 6). The motility was tested at both 30 and 37 °C (Fig. 6a) and the results showed a clear effect of the temperature but no effect of the stressosome mutation on the swimming of *V. vulnificus *. Haemolysin (Fig. 6b) and protease (Fig. 6c) production were assessed at 37 °C on LBN supplemented with sheep blood and skim milk, respectively. Both *V. vulnificus* wild-type and ΔRSTX showed degradation of the substrates, indicating that they are actively producing and secreting both haemolysin and protease. Although precise quantification is not possible using this method, no difference was observed between the two.

This set of experiments showed that the stressosome is not involved in motility and exoenzyme production in nutrient-rich media.

## Discussion

In this work, we focused on the phenotypic characterization of a *V. vulnificus* stressosome mutant lacking the upstream module of the stressosome genetic locus. This includes the genes encoding the three proteins forming the stressosome (VvRsbR, VvRsbS and VvRsbT) and the phosphatase VvRsbX [51, 52]. We decided to perform this characterization in the rich medium LBN, which is one of the most commonly used media in studies of the physiology of *V. vulnificus *. We focused on environmental stress responses, as these are the route of stressosome-mediated SigB activation in *B. subtilis* [20].

We first confirmed the presence of stressosome mRNA in wild-type bacteria grown to stationary phase in LBN, as the expression of these genes has been previously shown only in ASW [26] and minimal medium [28]. We also investigated the possible co-transcription of the upstream and downstream modules and we confirmed that products of co-transcription were present. This, together with their co-localization on chromosome 2 of *V. vulnificus *, supports the hypothesis that the two modules are functionally related and the downstream TCS could be the regulatory output of the stressosome in this bacterium.

Following this, construction of the knockout mutant using a classical mutagenesis protocol in a rifampicin-resistant parental background led to the realization that pleiotropic effects of *rpoB* mutations causing rifampicin resistance could interfere with stress response and virulence characterization of *V. vulnificus *. For this reason, we successfully optimized a new mutagenesis protocol that allowed construction of the mutant in a wild-type background due to the use of an auxotrophic *E. coli* donor strain. The mutant constructed in the wild-type background was then utilized for extensive phenotypic characterization in comparison to the wild-type strain. This started with growth characterization in LBN. We confirmed that there were no growth differences between the ΔRSTX mutant and the wild-type in the absence of stress, as expected, and focused on the effects of hypoxia and high temperature stresses on growth. Differences between the two strains did not emerge and the effects of hypoxia and high temperature were comparable between the two strains, despite the hypothesized role of the stressosome as an oxygen sensor [28], thus highlighting the media-specific role of the stressosome in *V. vulnificus *. Due to the predicted role of the stressosome in stress sensing and stress response, the next step of this phenotypic characterization was the assessment of stress survival and tolerance of the stressosome-lacking mutant. Survival was assessed in the presence of several stresses, including acid, ethanol, oxidative and temperature stress, and once again similar effects were observed for the wild-type and the stressosome mutant. Stress tolerance, defined as the ability to grow in the presence of sub-lethal stresses, was also tested and no effects of the stressosome were observed, even when stresses were combined, i.e. NaCl and hypoxia stress. Moreover, the lack of the stressosome did not affect the ability of *V. vulnificus* to swim and produce enzymes relevant to the infection process. According to these results, the presence of the stressosome genes confers no advantage to the human pathogen *V. vulnificus* in growth, stress tolerance and stress survival in LBN. While this lack of detectable influence on growth and survival in the presence of sub-lethal and lethal stresses was surprising considering the well-characterized role of the stressosome in Gram-positive bacteria [19, 24], the non-essential function of the stressosome does correlate with its presence in only 44 % of sequenced *V. vulnificus* isolates.

Recent research indicates that multiple triggers are required for stressosome expression and activation in *V. vulnificus *, including starvation, O_2_ limitation, iron limitation and acidity [28, 30]. Our data may suggest that this combination of triggers does not occur in nutrient-rich LBN. Studies demonstrating activity of the stressosome have been conducted in Fe-limited and nutrient-limited chemically defined medium (CDM). In these media maximum transcription and expression of the stressosome occurred in late-log/stationary phase, with lower levels in exponential phase, coinciding with glucose depletion and decreased pH [28, 30]. In CDM, stressosome mutants were more resistant to acid stress and displayed decreased motility, neither of which we observed in LBN, even during anaerobosis [30]. Furthermore, starvation and oxygen limitation were shown to trigger stressosome-dependent alterations in the proteome, implicating the stressosome as an O_2_ sensor [28]. Comparative proteomics revealed that absence of the stressosome resulted in downregulation of 157 proteins and upregulation of 148 proteins as compared to the wild-type *V. vulnificus *, indicating the importance of the stressosome in maintaining appropriate protein expression in oxygen-restricted conditions in CDM.

Therefore, the experimental conditions used here may not be compatible with stressosome activation despite the presence of the locus transcripts. A basal level of stressosome expression may occur as a means for the bacterium to monitor and survey for the appearance of stress signals. This could be seen as being analogous to the role of Toll-like receptors in eukaryotic cells acting as surveillance mechanisms for pathogen-associated and damage-associated molecular patterns that upon recognition and activation initiate signalling cascades, leading to inflammation and other cellular responses. Previous works have shown that the stressosome genes are expressed in ASW at higher levels than in human serum [26] and have shown a potential role in oxygen-sensing and iron metabolism regulation in minimal medium [28], indicating that the stressosome-mediated response could be naturally active in a nutrient-deficient environment rather than in rich media. Future studies in minimal, nutrient-depleted media would further characterize the activation pathway and the role of the stressosome as a regulatory complex in a Gram-negative human pathogen.

## Supplementary Data

Supplementary material 1
